# Induced Overexpression of Connexin43 in Astrocytes Attenuates the Progression of Experimental Temporal Lobe Epilepsy

**DOI:** 10.1007/s11064-025-04558-w

**Published:** 2025-09-18

**Authors:** Oussama Kherbouche, Lukas Henning, Pia Niemann, Caroline Geisen, Gerald Seifert, Christian Henneberger, Bernd K. Fleischmann, Christian Steinhäuser, Peter Bedner

**Affiliations:** 1 Institute of Cellular Neurosciences I, Medical Faculty, University of Bonn, Bonn, Germany; 2 Institute of Physiology I, Medical Faculty, University of Bonn, Bonn, Germany; 3 German Center for Neurodegenerative Diseases (DZNE), Bonn, Germany; 4 Present Address: Klinik für Neurologie, KRH Klinikum Agnes Karll Laatzen, Hannover, Germany; 5 Present Address: Department of Epileptology, University Hospital Bonn, Bonn, Germany

**Keywords:** Connexin 43, Gap junction coupling, Temporal lobe epilepsy, Electrophysiology, EEG, Hippocampal sclerosis

## Abstract

Astrocytic gap junctional communication plays a critical role in regulating neuronal activity and network synchronization, yet its precise contributions to brain function and the pathogenesis of neurological disorders remains incompletely understood. To address this, we generated a transgenic mouse line with inducible, astrocyte-specific overexpression of the gap junction protein connexin43 (Cx43). In these mice, hippocampal astrocytes exhibited markedly elevated Cx43 protein levels and a ~ 20% increase in intercellular gap junction coupling. Enhanced coupling was accompanied by a reduction in astrocytic cell volume and branching, without affecting passive membrane properties or astrocyte density in the hippocampus. Cx43 overexpression had no detectable impact on adult neurogenesis in the dentate gyrus, nor did it alter hippocampal synaptic efficacy or plasticity. Notably, in a mouse model of temporal lobe epilepsy with hippocampal sclerosis, astrocytic Cx43 overexpression attenuated chronic epileptic activity and the extent of sclerosis, supporting an antiepileptic role of the astroglial network. Collectively, these findings enhance our understanding of the functional relevance of astrocytic gap junction coupling in health and disease, with potential implications for the design of new treatment strategies.

## Introduction

Astrocytes in the mammalian brain are extensively interconnected through gap junction (GJ) channels, forming large-scale functional networks that overlap and interact with neuronal networks. This syncytial organization is crucial for the ability of astrocytes to regulate neuronal activity and enables the coordinated control and synchronization of large neuronal populations [1, 2]. GJ channels allow intercellular diffusion of ions and small molecules of up to ~ 1 kDa, such as nucleotides, second messengers, neurotransmitters, and energy metabolites. They are composed of transmembrane proteins of the connexin (Cx) family. Six Cx subunits oligomerize to form a hemichannel (connexon), which docks with a hemichannel from an adjacent cell to form a complete intercellular channel [1]. In the hippocampus, astrocyte-to-astrocyte communication is primarily mediated by Cx43 channels and, to a lesser extent, by channels composed of Cx30 [3]. Cx43 has four transmembrane domains, two extracellular loops, a cytoplasmic loop, and intracellular N- and C-terminal domains [4]. The C-terminal domain of Cx43 exerts a central regulatory function by harboring multiple protein–protein interaction motifs and phosphorylation sites for various kinases, which regulate channel gating, intracellular trafficking, protein turnover, and recruitment of scaffolding proteins [5].

Most information on the role of astrocytic networks in the healthy and diseased brain has been obtained from mice with coupling-deficient astrocytes. Under physiological conditions, a consistent finding across different models with impaired astrocytic coupling, including Cx30 and Cx43 single knockout mice [6, 7], constitutive and inducible astrocyte-specific Cx30/Cx43 double KO (dKO) mice [8, 9], as well as mice with virally expressed dominant negative Cx43 in astrocytes [10], is a reduction in long-term potentiation (LTP). Further findings from dKO mice included impaired K^+^ and glutamate buffering [8, 11], prolonged cell swelling during neuronal activity [8], reactivity of astrocytes and microglia [9], increase in excitatory synaptic transmission [8, 9], altered neuronal excitability (increased in constitutive and reduced in inducible dKO mice [8, 9]), impaired supply of energetic metabolites to neurons [12], and reduced adult neurogenesis in the dentate gyrus (DG) [13].

Although not yet fully understood, a growing body of evidence implicates disturbances in the astrocytic network as critical factors in epileptogenesis and disease progression [14, 15]. We have previously shown that astrocytes in post-surgical hippocampal specimens from temporal lobe epilepsy (TLE)-patients with hippocampal sclerosis (HS) completely lack GJ coupling [14]. In an experimental model of TLE, we reproduced this pathology and demonstrated that astrocyte uncoupling, along with impaired K⁺ clearance, precedes neuronal death and the emergence of spontaneous seizures, suggesting a causative role in epileptogenesis [14]. Furthermore, studies in both human and experimental TLE revealed that uncoupling is not attributable to reduced Cx expression, but to subcellular redistribution and altered phosphorylation of Cx43 [16]. Recent evidence suggests that these changes are driven by soluble TNFα of microglial origin [17].

In hippocampal slices from Cx30/Cx43 dKO mice spontaneous epileptiform events and a reduced threshold for the generation of epileptic activity were reported [11]. Consistent with that, dKO animals showed higher seizure frequencies and interictal spike activity in a chronic TLE model [18]. In contrast, another study showed that dKO mice are less susceptible to pentylenetetrazol (PTZ)-induced hyperexcitability [19]). Furthermore, neither constitutive nor inducible, astrocyte-specific dKO mice showed spontaneous seizures or abnormal EEG activity in vivo [9, 19]. However, in contrast to the situation in human and experimental TLE, dKO mice not only lack intercellular coupling but also the Cx proteins, thereby excluding potential pro-epileptic non-channel or hemichannel functions of Cxs [6, 20].

Overall, the role of the astroglial network for normal brain function and in the development and progression of TLE is not yet fully understood. To further elucidate this clinically relevant topic, we generated mice with inducible overexpression of Cx43 in astrocytes and investigated consequences of enhanced astroglial coupling on hippocampal function and epileptogenesis.

## Materials and Methods

### Animals

**Fig. 1:**
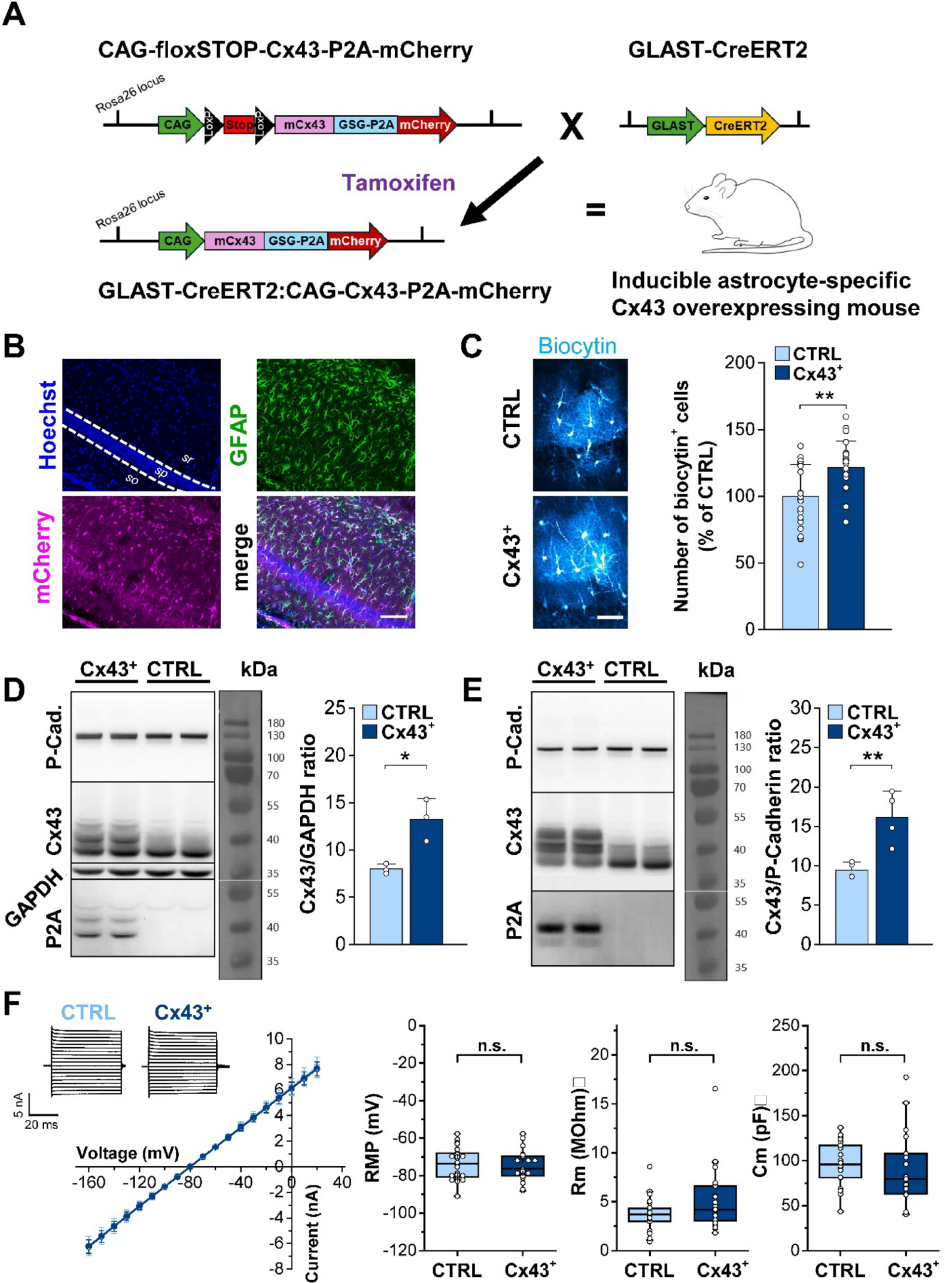
Generation and characterization of the inducible astrocyte-specific Cx43 overexpressing (Cx43^+^) mouse line. **A** Scheme illustrating the generation of tamoxifen-inducible astrocyte-specific Cx43 overexpressing mice (GLAST-CreERT2:CAG-floxSTOP-Cx43-P2A-mCherry). Mice carrying the CAG-floxSTOP-Cx43-P2A-mCherry cDNA in the Rosa26 locus were crossed with heterozygous mice expressing a tamoxifen-inducible CreERT2 recombinase under the control of the astrocyte-specific GLAST promoter. Recombination was induced at p30 by i.p. injection of tamoxifen (2 mg/kg/day) for five consecutive days. Upon Cre recombinase activity, the loxP-flanked stop sites are excised and exogenous Cx43, tagged at the C-terminus with 21 amino acids of P2A, and mCherry are expressed under control of the CAG promoter. **B** Efficiency and specificity of recombination in hippocampal CA1 astrocytes of GLAST-CreERT2:CAG-Cx43-P2A-mCherry mice was assessed by Hoechst (blue), GFAP (green) and mCherry (red) triple staining. Scale bar: 100 μm. **C** Representative maximum intensity projections (MIPs) showing the efficiency of interastrocytic coupling, assessed by the extent of biocytin diffusion from a single cell, filled with the tracer through the patch pipette during a 20 min whole cell recording (left). Scale bar: 50 μm. Cx43^+^ mice displayed a ~ 20% higher number of biocytin-positive cells in the hippocampus (right). *n* = 29 and 27 cells from Cx43⁺ and CTRL mice, respectively, *N* = 5 animals/genotype. **D** Whole cell lysates from hippocampi were subjected to SDS-PAGE followed by Western blotting. Cx43, GAPDH, P-cadherin and P2A proteins were evaluated (left). GAPDH served as a loading control. Bar graphs (right) show densitometric analyses of the WBs. Cx43 was upregulated in Cx43⁺ mice. P2A was exclusively detected in Cx43⁺ mice. *n* = 3 samples/mouse, *N* = 3 mice/genotype. **E** Plasma membrane associated-proteins isolated from hippocampi were subjected to SDS-PAGE followed by Western blotting. P-cadherin served as a loading control (left). Quantification confirmed that new Cx43 protein was inserted into the plasma membrane of astrocytes in Cx43⁺ mice (right). *n* = 2 samples/mouse, *N* = 4 mice/genotype. **F** Passive membrane properties of astrocytes overexpressing Cx43. Traces on the top left show representative whole-cell current patterns of astrocytes in the CA1 *str. rad.* of the hippocampus of Cx43^+^ and control mice, elicited by voltage steps ranging from − 160 to + 20 mV (10 mV increments). The graph below shows the corresponding current–voltage (I-V) relationship. Graphs on the right display passive membrane properties. Neither RMP nor Rm or Cm differed between the genotypes. Data are presented as box plots with median (central line), quartiles (25% and 75%; box) and whiskers extending to the highest and lowest values within 1.5 times the interquartile range. *n* = 21 and 26 cells from Cx43⁺ and CTRL mice; *N* = 5 mice/genotype. *p < 0.05, **p < 0.01; n.s., not significant (*p* > 0.05).

To generate Cx43 overexpressing (Cx43^+^) mice, we integrated a DNA fragment, consisting of murine Cx43 cDNA, the GSG-P2A sequence, and mCherry cDNA under the control of the CAG promoter into the mouse Rosa26 locus. Transcription of the expression cassette was inhibited by a loxP-flanked stop cassette until it was removed by Cre recombinase activity. Functionality of the construct was extensively validated in cell culture [21]. To achieve astrocyte-specific overexpression of Cx43, mice carrying the CAG-floxSTOP-Cx43-P2A-mCherry cDNA in the Rosa26 locus were crossed with mice heterozygously expressing the tamoxifen-inducible form of Cre recombinase (CreERT2) in the locus of the astrocyte-specific glutamate transporter (GLAST) [22] (Fig. 1A). To induce site-specific recombination of loxP-flanked sequences, GLAST-CreERT2:CAG-floxSTOP-Cx43-P2A-mCherry mice received intraperitoneal (i.p.) injections of tamoxifen (Sigma-Aldrich, Steinheim, Germany) dissolved in sunflower seed oil (Sigma-Aldrich) and ethanol (EtOH) at a 1:10 ratio. To induce Cre-dependent recombination, four-week-old mice of both sexes received tamoxifen (2 mg/day; 100 µl per mouse) for 5 consecutive days. Experiments were performed 30–90 days later (age of the mice: 2–4 months). For most experiments, tamoxifen-injected GLAST-CreERT2 littermates served as the control group (CTRL). For EEG analysis, additionally GLAST-negative CAG-floxSTOP-Cx43-P2A-mCherry littermate CTRLs were used. As there were no significant differences in epileptiform activity between the two CTRL groups, the results were pooled.

Maintenance and handling of animals were performed according to EU and local governmental regulations. Experiments were approved by the North Rhine–Westphalia State Agency for Nature, Environment and Consumer Protection (approval number 81-02.04.2020.A420). All measures were taken to minimize the number of animals. Mice were kept under standard housing conditions (12 h/12 h light-dark cycle) with food and water provided ad libitum.

### Whole Cell Patch Clamp Recording and Biocytin Loading of Astrocytes

Mice were anesthetized with isoflurane (Piramal Healthcare, Morpeth, UK) and decapitated. Brains were quickly removed, and 200 μm-thick coronal slices were cut on a vibratome (VT1000S, Leica Microsystems, Wetzlar, Germany) in ice cold preparation solution containing (in mM): 87 NaCl, 2.5 KCl, 1.25 NaH_2_PO_4_, 25 NaHCO_3_, 7 MgCl_2_, 0.5 CaCl_2_, 25 glucose, 75 sucrose, equilibrated with carbogen to stabilize pH (5% CO_2_/95% O_2_, pH 7.4). After storage of slices (15 min, 35 °C) in preparation solution, slices were transferred to a solution containing (in mM): 126 NaCl, 3 KCl, 2 MgSO_4_, 2 CaCl_2_, 10 glucose, 1.25 NaH_2_PO_4_, 26 NaHCO_3_, gassed with carbogen (aCSF). To aid identification of astrocytes in the tissue, aCSF was supplemented with SR101 (1 µM, Sigma Aldrich, incubation 20 min, 35 °C) [23]. After SR101 staining, slices were transferred to aCSF and kept at room temperature (RT) for the duration of the experiments. For recordings, slices were transferred to a recording chamber and constantly perfused with aCSF. Patch pipettes fabricated from borosilicate capillaries with a resistance of 3–6 MΩ were filled with a solution containing (in mM): 130 K-gluconate, 1 MgCl_2_, 3 Na_2_-ATP, 20 HEPES, 10 EGTA and biocytin (0.5%, Sigma Aldrich) (pH 7.2, 280–285 mOsm). To analyze GJ coupling, whole-cell patch clamp recordings of SR101-positive astrocytes were performed during which astrocytes were filled with biocytin (20 min, RT). In addition to SR101 staining, astrocytes were identified by their characteristic morphology, passive current–voltage relationship, low input resistance and a resting membrane potential (RMP) close to the Nernst potential of K^+^. Current signals were amplified (EPC 8, HEKA Electronic, Lambrecht, Germany), filtered at 3–10 kHz, and sampled at 10–30 kHz (holding potential − 80 mV). Online analysis was performed with TIDA 5.25 acquisition and analysis software for Windows (HEKA) and Igor Pro 6.37 software (WaveMetrics, Lake Oswego, OR, USA). Voltages were corrected for liquid junction potentials. Only recordings matching the following criteria were included in the analysis: (i) RMP negative to −60 mV, (ii) membrane resistance (Rm) ≤ 10 MΩ, and (iii) series resistance ≤ 20 MΩ. After recording, slices containing biocytin-filled astrocytes were stored in 4% paraformaldehyde (PFA)-containing phosphate buffered saline (PBS) overnight at 4 °C and subsequently transferred into PBS and stored at 4 C until immunohistochemistry.

### Western Blotting

Mice were anesthetized using isoflurane (Piramal Healthcare) and subsequently decapitated. Brains were rapidly extracted, and bilateral hippocampi were carefully dissected and immediately placed on ice. Hippocampi were then stored at −20 °C until preparation. Whole-cell protein lysates were prepared by homogenizing mouse hippocampal tissue in a modified radioimmunoprecipitation assay (RIPA) buffer (50 mM Tris-HCl, pH 7.5; 1% Nonidet P-40; 0.25% sodium deoxycholate; 150 mM NaCl; 1 mM EDTA, pH 8.0) supplemented with 1 mM phenylmethylsulfonyl fluoride (PMSF) (Sigma-Aldrich, #P7626) and 1% Halt™ Protease and Phosphatase Inhibitor Single-Use Cocktail (Thermo Scientific, #78442). Plasma membrane proteins were isolated from hippocampal tissue using the Plasma Membrane Protein Extraction Kit (Abcam, #ab65400) according to the manufacturer’s protocol. Briefly, hippocampal tissue was homogenized in the provided buffer, followed by centrifugation at 11,000 × g for 30 min at 4 °C. The resulting pellet, which corresponded to total cellular membranes, was extracted and resuspended in two partially miscible polymer solutions of the kit in order to selectively extract plasma membrane proteins. The extracted final pellet containing plasma membrane proteins was dissolved in RIPA buffer, supplemented with PMSF and Halt™ Protease and Phosphatase Inhibitor Cocktail with the same concentrations. Total protein concentration of each sample was determined using the Pierce™ BCA Protein Assay Kit (Thermo Scientific, #23225), with an albumin standard used as a reference. Five µg of plasma membrane proteins and 30 µg of whole-cell lysates were taken and subjected to SDS-PAGE. After successful SDS-PAGE and protein transfer onto PVDF membranes, these were blocked with 5% non-fat milk in Tween^®^ 20 Detergent (TBST) for 30–60 min and incubated overnight at 4 °C with primary antibodies (rabbit anti-Cx43 (1:3000, custom-made [24]),; mouse anti-P-Cadherin (1:5000, Abcam, #ab6529); mouse anti-GAPDH (1:5000, Abcam, #8245, Cambridge, UK); rabbit anti-mCherry (1:3000, Abcam, #ab167453); mouse anti-2 A Peptide (3H4) (1:700, Novus Biologicals, #NBP2-59627). Following 3 washes in Tris-buffered saline with TBST, membranes were probed with horseradish peroxidase (HRP)-conjugated secondary antibodies for 1 h in 2.5% milk (donkey anti-rabbit IgG, HRP-linked F(ab’)₂ fragment (Amersham ECL™, #NA9340V); sheep antimouse IgG, HRP-linked F(ab’) fragment (Amersham ECL™, #NA9310V)). Protein bands were visualized using the GeneGnome XRQ™ Imaging System (Syngene, UK) after applying WesternBright Sirius Chemiluminescent Substrate (Advansta, #K-12043-D20). Densitometric analysis was performed using GeneTools (Syngene). For successive detection of multiple proteins on the same membrane, antibodies were stripped using Restore™ Western Blot Stripping Buffer (Thermo Scientific, #21059) for 15 min, followed by three washes with distilled water (5 min each), before re-incubation with new primary antibodies. Protein intensities were normalized to loading controls: P-cadherin for plasma membrane fractions and GAPDH for whole-cell lysates.

### Immunohistochemistry

Animals were deeply anaesthetized by i.p. injection with 100–120 µl of a solution containing 40 mg/kg ketamine (Ketamidor, WDT, Garbsen, Germany) and 0.3 mg/kg medetomidine (CP-Pharma, Burgdorf, Germany). After checking for hind paw reflexes, transcardial perfusion was applied with ice-cold PBS (30 ml) followed by 4% ice-cold PFA in PBS (30 ml). Brains were removed and stored for 24–48 h in 4% PFA-containing solution and subsequently in PBS at 4 °C until slicing. Brains were cut into 40 μm thick coronal slices using a Leica VT1200S vibratome (Leica Microsystems).

For antibody staining, free-floating slices were incubated at RT (2 h) with PBS containing 0.5% Triton X-100 (or 2% for biocytin-filled astrocyte staining) and 10% normal goat (NGS) to permeabilize membranes and block nonspecific epitopes. Subsequently, slices were incubated overnight with primary antibody solution on a shaker at 4 °C. The following primary antibodies were applied: mouse anti-GFAP (1:500, Millipore, #MAB360, Darmstadt, Germany), rabbit anti-GFAP (1:500, DAKO, Z0334, Hamburg, Germany), rabbit anti-s100ß (1:200, Abcam, ab41548), mouse anti-NeuN (1:300, Merck Millipore, MAB377), rabbit anti-Ki67 (1:500, Novocastra, NCL-Ki67p, Berlin, Germany), rabbit anti-mCherry (1:500, Abcam, ab167453). On the following day, slices were washed three times with PBS for 5–10 min each and subsequently incubated for 2 h at RT in PBS containing 2% NGS and secondary antibodies conjugated with goat anti-mouse Alexa Fluor^®^ 488, goat anti-rabbit Alexa Fluor^®^ 555 and goat anti-rabbit Alexa Fluor^®^ 647 (1:500 each, Invitrogen, Karlsruhe, Germany). For NeuN staining, slices were first incubated with a goat anti-mouse biotin antibody (1:500, Dianova, AB_2338557, Hamburg, Germany), followed by incubation with streptavidin-conjugated Cy3 antibody (1:300, Sigma Aldrich, S6402) for 1 h at RT. After three additional PBS washes (10 min each), nuclear staining was performed using Hoechst (1:200, diluted in dH₂O) for 10 min at RT. A final series of PBS washes (3 times, 10 min each) was conducted before slices were mounted on microscope slides using Aquapolymount (Polysciences, Heidelberg, Germany) and covered with coverslips. Prepared slides were stored at 4 °C until confocal imaging. Confocal imaging was conducted using a Leica SP8 laser scanning microscope (Leica Microsystems, Hamburg, Germany) at 8-bit resolution with objectives of 10× (Numerical Aperture [NA]: 0.4) and 40× (NA: 1.1) magnification. Images were acquired at a resolution of 1024 × 1024 pixels, with a scanning speed of 400 Hz and a pinhole size of 1 airy unit (AU). Fluorescent signals were detected using either standard photomultiplier tubes or hybrid detectors. Laser and detector settings were kept consistent across all images to ensure comparability. Z-stacks were recorded at depth intervals of 1 μm.

### Quantification of Immunostaining

Image analyses were conducted using FIJI (imageJ). Astrocyte cell counting in the hippocampal CA1 region was performed manually using high-resolution images acquired at 40× magnification (NA: 1.1). A region of interest (ROI) of 180 × 180 × 20 µm^3^ was delineated within the *stratum radiatum* (*str. rad.*) and extracted for analysis. Only S100β-positive astrocytes containing a Hoechst-positive nucleus were included in the analysis. Astrocytes intersecting the right and upper edges of the image were counted, whereas those at the left and bottom edges were omitted. In the depth dimension, only astrocytes intersecting the upper focal planes were included, while those extending into deeper sections were excluded. The final astrocyte count was averaged across images.

Quantification of GFAP-immunoreactivity was also performed in ROIs of 180 × 180 × 20 µm^3^ within the CA1 *str. rad.* in images taken at x40 magnification. Background intensity was measured across different areas of various layers, averaged, and subtracted from the GFAP image to obtain a corrected signal. GFAP immunoreactivity was then measured within the ROI for each layer and averaged across all layers.

For astrocyte 3D quantification, 2 to 3 GFAP^+^ cells were randomly selected and cropped from 30 μm-thick image stacks obtained from each mouse to generate single-cell images. To analyze astrocyte 3D morphology, we utilized the MotiQ plugin for Fiji [25]. Single-cell images were binarized by applying an automated intensity threshold (Otsu) to 0.5-scaled maximum intensity projections (MIPs) of the original images. Binary single-cell images were further processed by applying a size filter to exclude particles smaller than 150 voxels. Subsequently, 3D reconstructions and skeletonized models were generated to quantify process number, average process length, and the ramification index. The ramification index represents the ratio of the cell’s surface area to the surface area of a sphere with an equivalent volume and is calculated using the following equation:

$$\:Ramification\:index=\frac{\text{C}\text{e}\text{l}\text{l}\:\text{S}\text{u}\text{r}\text{f}\text{a}\text{c}\text{e}\:\text{A}\text{r}\text{e}\text{a}}{\begin{array}{c}4\pi\:{\left(\frac{3\times\:Cell\:Volume}{4\pi\:\:}\right)}^{2/3}\\\:\end{array}}$$

Analysis of S100β-positive cell volumes was performed using the MotiQ plugin, based on the Intermodes automatic intensity threshold applied to 0.5-scaled MIPs and a filter excluding particles smaller than 100 voxels. 3D reconstructions failed in *n* = 5 cells and those were removed from the final dataset. For the assessment of neurogenesis, images were acquired using a 10× magnification objective in the DG. Ki67-positive cells within the subgranular zone (SGZ) were quantified and added up using a systematic 1-in-5 series sampling approach, yielding a total of 18 analyzed sections (corresponding to 36 DG sections) covering the entire rostrocaudal extent of the DG, in accordance with the methodology described in [26]. Numbers of Ki67-positive cells per section are given.

Coupling efficiency was assessed by manually counting biocytin-positive cells using the cell counter plugin in FIJI. Cell counts were compared between both genotypes and between kainic acid (KA) injected (ipsilateral) and non-injected (contralateral) hemispheres. The number of biocytin-positive cells was determined by two independent experimenters and values were averaged across both counts before statistical analysis.

Quantification of the extent of HS was performed as previously described [17]. One month following KA injection, mice were decapitated and underwent immunohistochemical analysis as described above. To ensure consistency across samples, all slices were selected from the same anatomical level, close to the KA injection site. Three parameters were quantified to assess HS: CA1 pyramidal neuron number, granule cell dispersion (GCD) in the DG, and the extent of astrogliosis in the CA1 *str. rad.*. Quantification of CA1 pyramidal neurons was conducted using an automated spot detection algorithm in IMARIS 8.0 (Bitplane, Zurich, Switzerland). The number of neurons was determined within 360 × 120 × 30 μm³ ROIs positioned in the CA1 pyramidal layer, directly above the highest point of the DG granule cell layer. The other parameters of HS were analyzed using FIJI. Astrogliosis was quantified by measuring GFAP immunoreactivity in ROIs of 320 × 100 × 23.3 µm^3^ located in the CA1 *str. rad.* just above the highest curvature of the DG granule cell layer as described above. GCD was assessed using MIP z-stacks. A reference line extending from the hilus tip to the curvature of the upper blade of the DG was defined (Fig. 5A). At the endpoint of this line, a perpendicular line was drawn spanning from the upper to the lower blade of the DG. A second perpendicular line was positioned at the midpoint of the reference line. GCL width was measured at four predefined positions (T1, T2, T3, T4): T1 and T2 were measured along the first perpendicular line at the upper and lower blades, respectively, while T3 and T4 were measured along the second perpendicular line at the corresponding upper and lower blades. The mean of these four measurements provided a quantitative estimate of GCD extent.

### Field Potential Recordings

Experiments were conducted in an interface chamber. Slices (300 μm) were continuously perfused with carbogenated aCSF (35 °C; 2.3 ml/min) containing (in mM): 132 NaCl, 3 KCl, 2 MgCl₂, 2 CaCl₂, 10 glucose, 1.25 NaH₂PO₄, and 20 NaHCO₃. Electrodes were positioned under optical guidance using manually controlled micromanipulators. Fiber stimulation was delivered using a bipolar concentric electrode (CBARC75; FHC). Stimulus intensity was controlled using an isolated current stimulator (DS3, Digitimer Ltd.). Signals were pre-amplified and filtered (high-pass: 0.1 Hz, low-pass: 20 kHz; EXT-02B, npi), with 50 Hz interference eliminated using a HumBug noise eliminator (Quest Scientific Instruments, Inc.). Signals were sampled at a frequency of 10 kHz (NI USB-6221, National Instruments). Recordings were obtained via a Teflon-coated silver wire with a chlorinated tip. Schaffer collaterals were stimulated at the interface between the *str. rad.* of CA3 and CA1, while extracellular recordings were obtained using patch pipettes (see above) filled with extracellular solution placed in the CA1 *str. rad*. Data was recorded using WinWCP 4.6.1 software. Postsynaptic responses were quantified by measuring the field excitatory postsynaptic potentials (fEPSP) slope during the linear rising phase. After electrode placement, slices were allowed to stabilize for 20 min. Before recordings, a stimulus-response curve was registered using increasing stimulation intensities ranging from 25 to 400 µA. Each stimulus intensity was applied three times, and the mean values were calculated to assess the initial slope of the fEPSPs. The stimulus intensity leading to half maximum fEPSPs was used as the stimulation intensity for further experiments. After selecting the stimulation intensity, baseline paired-pulse recordings (inter-stimulus interval: 50 ms) were obtained every 15 s for 10 min before induction of synaptic long-term potentiation (LTP). LTP was induced by theta burst stimulation (TBS), consisting of 8 stimulus trains delivered at 5 Hz (each train containing 4 pulses at 100 Hz), which was done three times in total with 1-min intervals. Following TBS, baseline paired-pulse stimulation continued for an additional 30 min every 15 s. Data analysis was conducted using Clampfit (V. 10.3; Molecular Devices, LLC). fEPSP slopes were normalized to the average fEPSP slope recorded during the initial 10 min (baseline). Fiber volley amplitudes were compared across all experiments, revealing no significant differences. Data were excluded if baseline variability exceeded 10%. The magnitude of LTP was assessed using the normalized response 25–30 min after TBS. Paired-pulse ratios (PPR) were assessed by calculating the ratio of the second fEPSP slope relative to the first one.

### Unilateral Intracortical KA Injection and Implantation of Telemetric EEG Transmitters

We employed the TLE-HS animal model as previously described [14, 17]. Briefly, mice were anesthetized via i.p. injection of a mixture of medetomidine (Cepetor, CP-Pharma, Burgdorf, Germany; 0.3 mg/kg) and ketamine (Ketamidor, WDT, Garbsen, Germany; 40 mg/kg) and positioned in a stereotaxic frame equipped with a manual microinjection unit (David Kopf, Tujunga, CA, USA). A total of 70 nl of a 20 mM KA solution (Tocris, Bristol, UK), dissolved in 0.9% sterile NaCl, was stereotaxically injected into the neocortex directly above the right dorsal hippocampus. Injection coordinates were 2 mm posterior to bregma, 1.5 mm lateral from the midline, and 1.7 mm below the skull surface. Immediately after KA injection, two small burr holes were drilled 1 mm posterior to the injection site and 1.5 mm lateral from the midline to place two monopolar leads for EEG recording. Telemetric transmitters (TA10EA-F20 or TA11ETA-F10; Data Sciences International, St. Paul, MN, USA) were implanted subcutaneously into the right abdominal flank, and the leads were inserted 1 mm deep into the cortex. Leads were fixed to the skull using superglue and secured with dental cement. Afterward, the scalp incision was sutured, and anaesthesia was reversed using atipamezole (Antisedan, Orion Pharma, Hamburg, Germany; 300 µg/kg, i.p.). To manage postoperative pain, mice received carprofen (Rimadyl, Pfizer, Karlsruhe, Germany) for three days. Additionally, 0.25% enrofloxacin (Baytril, Bayer, Leverkusen, Germany) was administered via drinking water to prevent infection. Following surgery, animals were returned to clean cages and placed on individual radio receiver plates (RPC-1; Data Sciences International, New Brighton, MN, USA), which transmitted data from the implant to a computer running Ponemah software (v5.2, DSI). Continuous EEG monitoring (24 h/day, 7 days/week) was initiated immediately after transmitter implantation and maintained for 28 days post *status epilepticus* (SE; defined as series of convulsive seizures lasting up to 6 h, Bedner et al., 2015) induction.

### EEG Data Analysis

NeuroScore 3.4.0 software (DSI) was used for EEG analysis as described previously [17]. Briefly, recordings were high-pass filtered at 1 Hz, and the number of seizures, their duration and spike numbers were determined using the spike train analysis tool implemented in NeuroScore based on the following parameters: threshold value range = 10x or 7.5x the standard deviation (during SE or the chronic phase, respectively) of the baseline (i.e., activity during artifact- and epileptiform-free epochs four weeks after SE) – 1 mV, spike duration = 0.1–50 ms, spike interval = 0.1–2.5 s, minimum train duration = 30 s, train join interval = 1 s, minimum number of spikes = 50. All detected seizures were additionally verified by manual review. Seizure number and duration were analyzed only during the first hour of SE, as individual seizures were clearly distinguishable only in this timeframe. For spectral analysis, EEG data were subjected to fast Fourier transform (FFT) and the absolute power of the frequency bands δ (0.5–4 Hz), γ (30–50 Hz) and total (0.5–50 Hz) was calculated in 10 s epochs and normalized to baseline activity (power during artifact- and epileptiform-free epochs four weeks after SE) prior to statistical analysis. Spectral and spike analyses were conducted during the first 4 h of SE and during two separate 24 h periods in the chronic phase, typically on days 21 and 28 post SE.

### Statistical Analysis

Statistical analyses were conducted with OriginPro (OriginLab Corporation, Northampton, MA, USA) and R software (R version 4.3.1). Data are presented as mean ± SD or as box plots displaying median (center line), interquartile range (IQR; 25th–75th percentile), and whiskers extending to the lowest and highest values within 1.5× IQR. Before statistical testing, normality was assessed using the Shapiro-Wilk test. Levene’s test was applied to evaluate the homogeneity of variance across groups. For parametric data with equal variances, a two-sample Student’s t-test was performed. Student’s t-test with Welch’s correction was used in case of unequal variances. For non-parametric data, the Mann-Whitney U test was employed. In case of a deviation from normality of multifactorial datasets, data were transformed according to Tukey’s ladder of powers [27]. Two-way ANOVA with Tukey post hoc test was used for multi-factorial analyses. For datasets with multiple measurements per mouse, linear mixed-effect models (LMMs) were applied, with *Genotype* (WT vs. Cx43^+^) as a fixed effect and mouse identity as a random effect. For sclerosis analyses, data were analyzed using LMMs with *Genotype* and *Side* as fixed factors and slice identity as a random effect to account for paired ipsilateral/contralateral measurements within each slice. Planned post hoc contrasts of estimated marginal means were used to test ipsi–contra differences within each genotype and genotype differences on the ipsilateral side, with p-values adjusted using the Holm method. Repeated measures ANOVA was used to analyze astrocytic current–voltage relationships and stimulus-response fEPSP data. Kaplan–Meier estimates were compared using a log-rank test. In all cases, *N* refers to the number of mice, and n to the number of analyzed slices/cells/samples. Statistical significance was set at *p* < 0.05.

## Results

### Characterization of Cx43 Overexpressing Mice

To gain further insight into the role of astroglial networks in brain physiology and the pathogenesis of epilepsy, we used transgenic mice conditionally overexpressing Cx43 in astrocytes (Fig. 1A). We tested recombination efficiency and cell-type specificity of Cx43 overexpression in GLAST-CreERT2:CAG-Cx43-P2A-mCherry (Cx43^+^) mice by immunohistochemical Hoechst/GFAP/mCherry staining (Fig. 1B). Quantification of staining revealed that 89 ± 6.3% (mean ± SD) of GFAP^+^ hippocampal astrocytes co-expressed mCherry, while almost all (98 ± 1.2%) mCherry^+^ cells also exhibited GFAP immunoreactivity (*n* = 17 slices, *N* = 4 mice), indicating high recombination efficiency and astrocyte specificity (quantitative data not shown). To determine whether Cx43 overexpression increases coupling efficiency, we assessed the spread of biocytin after tracer filling of individual astrocytes in acute hippocampal slices. The results show that the number of biocytin-positive astrocytes was approximately 20% higher in Cx43^+^ vs. CTRL mice (CTRL: 154.7 ± 37 vs. Cx43^+^: 189 ± 27.9 biocytin-positive cells, *p* = 0.0015, t-test) (Fig. 1C). In a parallel approach, we performed Western blot (WB) analysis to compare hippocampal Cx43 protein levels between Cx43^+^ and CTRL mice. Total protein lysates were immunoblotted and the membranes probed with antibodies directed against Cx43, P2A, P-cadherin and GAPDH (Fig. 1D). GAPDH was used as a loading control, while P-cadherin was included to test its suitability as a loading control in WB of plasma membrane protein fractions (see below). As expected, P2A could only be detected in lysates from Cx43^+^ mice, but not in those from the control group. In addition, anti-Cx43 antibody yielded additional bands corresponding to the different phosphorylation states of P2A-labeled Cx43 protein that overlapped with the P1 and P2 bands of the endogenous Cx43 protein. This overlap made a separate quantification of the phosphorylaion bands difficult. Therefore, Cx43 expression levels were quantified without distinguishing between individual phospho-bands. GAPDH-normalized total Cx43 protein ratios were significantly higher in Cx43^+^ vs. CTRL mice (CTRL: 7.97 ± 0.53 vs. Cx43^+^: 13.2 ± 2.2; *p* = 0.016, t-test, Fig. 1D). P-cadherin levels did not differ between both groups. Consistently, plasma membrane-associated Cx43 protein levels, normalized to P-cadherin, were also elevated in Cx43^+^ mice (CTRL: 9.47 ± 0.98 vs. Cx43^+^: 16.16 ± 1.67; *p* = 0.008, t-test, Fig. 1E). Together, these data show that astrocytes in the hippocampus of GLAST-CreERT2:CAG-Cx43-P2A-mCherry mice exhibit not only increased levels of total and membrane-associated Cx43 protein but also enhanced coupling efficiency.

To examine whether overexpression of Cx43 affects passive membrane properties of astrocytes, RMP, Rm and membrane capacitance (Cm) were assessed by whole-cell patch clamp recordings of astrocytes located in the CA1 *str. rad.*. No differences in current–voltage (I-V) relationships (*p* = 0.65, two-way repeated measures ANOVA) or in any of the passive membrane parameters were observed between astrocytes in Cx43^+^ and CTRL mice (RMP, CTRL: −73.55 ± 8.52 mV vs. Cx43^+^: −74.5 ± 8.16 mV, *p* = 0.7, t-test; Rm, CTRL: 3.64 ± 1.68 vs. Cx43^+^: 5.28 ± 3.4, *p* = 0.052, t-test with Welch correction; Cm, CTRL: 94 ± 22.7 vs. Cx43^+^: 83.76 ± 40.75, *p* = 0.57, t-test with Welch correction; Fig. 1F). Thus, Cx43 overexpression had no influence on passive membrane properties of hippocampal astrocytes.

### Altered Astrocyte Morphology but Unchanged Adult Neurogenesis in Cx43^+^ Mice

**Fig. 2:**
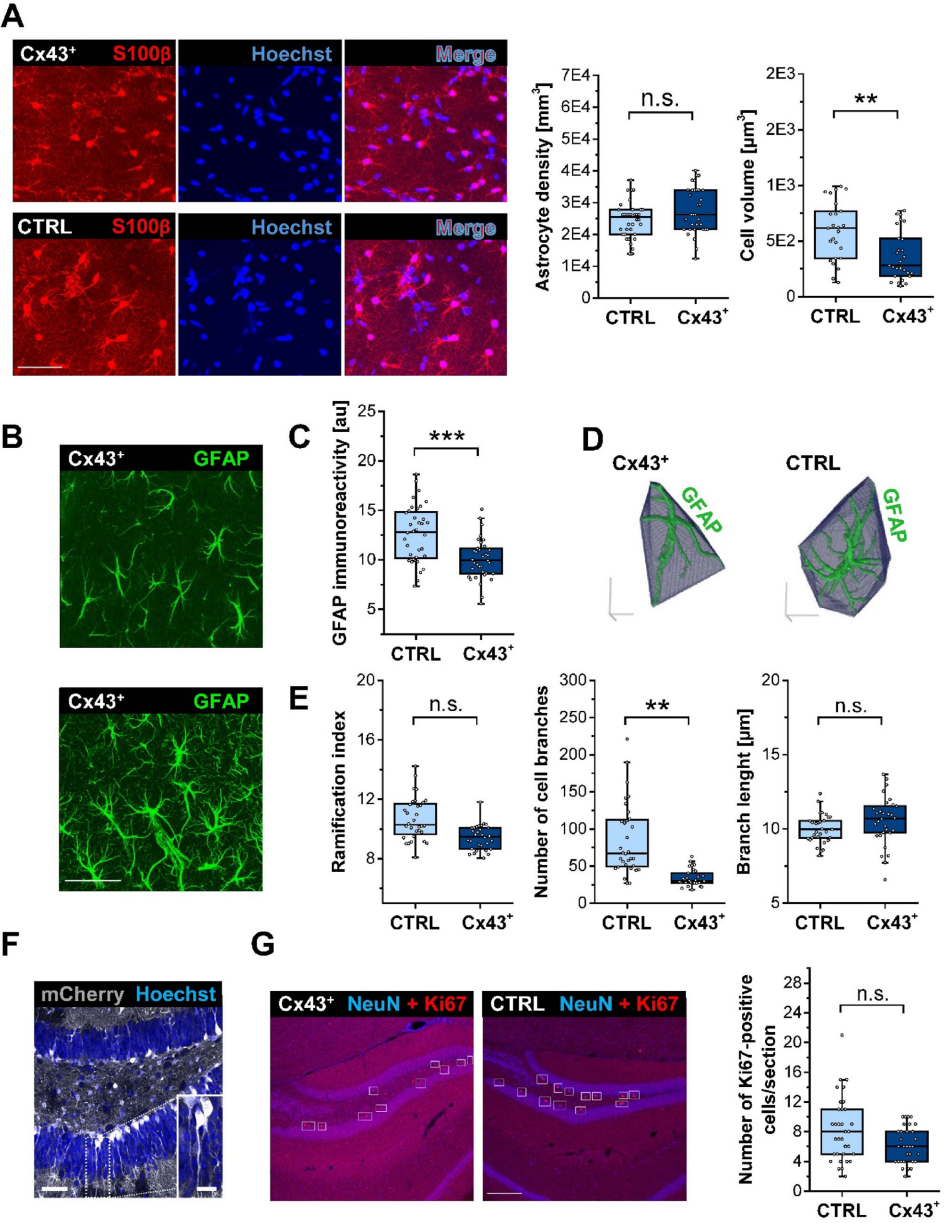
Cx43 overexpression affected the morphology CA1 astrocytes while its density and adult neurogenesis in the DG remained unchanged. **A** Representative MIPs displaying S100β-Hoechst double staining in the *str. rad.* of the hippocampal CA1 region. Scale bar: 50 μm (left). Manual counting of S100β^+^/Hoechst^+^ cells within ROIs of 180 × 180 × 20 μm³ revealed no difference in astrocyte density between CTRL and Cx43^+^ mice. *n* = 36 (CTRL) and 33 slices (Cx43^+^); *N* = 6 animals per genotype. Quantification of cell volumes in 3D reconstructions of individual S100β-positive astrocytes revealed significantly reduced astrocyte volumes in Cx43^+^ compared to CTRL mice. *n* = 25 (CTRL) and 27 (Cx43^+^) cells; *N* = 6 animals per genotype. **B** Representative confocal images of GFAP^+^ astrocytes in the *str. rad.* of the hippocampal CA1 region. Scale bar: 50 μm. **C** Quantification of GFAP fluorescence intensity within a ROI of 180 × 180 × 20 μm³ in the hippocampal *str. rad.* region revealed a lower overall GFAP immunoreactivity in Cx43^+^ mice. *n* = 36 (CTRL) and 35 slices (Cx43^+^); *N* = 6 animals per genotype. **D** Representative 3D MotiQ-rendered structures of individual GFAP^+^ astrocytes. Scale bars: 20 μm. Reconstructed astrocytes were obtained from the same images used for GFAP fluorescence intensity analysis. **E** Quantitative analyses of the ramification index, average branch number and length of GFAP^+^ cells indicated lower astrocyte cell volume and branch numbers in Cx43^+^ vs. control mice. *n* = 32 (CTRL) and 30 cells (Cx43^+^); *N* = 6 animals per genotype. **F** Representative MIPs showing mCherry (gray)-Hoechst (blue) double staining in the DG of GLAST-CreERT2:CAG-Cx43-P2A-mCherry mice. Image revealed strong mCherry expression in cells with morphological characteristics of RG-like cells in the SGZ of the DG. The inset shows an enlarged view of the dashed box area. Scale bar: 50 μm and 10 μm (inset). **G** Confocal images of the DG of Cx43^+^ and control mice illustrating NeuN (blue)-Ki67 (red) double immunostaining. Scale bar = 200 μm. Quantitative evaluation of Ki67^+^ cells in the SGZ of the DG revealed no difference between genotypes, suggesting that adult neurogenesis was not affected by Cx43 overexpression. *n* = 33 (CTRL) and 31 (Cx43^+^) sections; *N* = 6 mice per genotype. Data are presented as box plots with median (central line), quartiles (25% and 75%; box) and whiskers extending to the highest and lowest values within 1.5 times the interquartile range (IQR). Open circles represent individual data points. *p < 0.05; n.s., not significant (*p* > 0.05).

Lack or reduction of astrocyte GJ coupling induces astrocyte hypertrophy and reactivity [8, 9]. To assess whether enhanced coupling between astrocytes impacts their morphology, we conducted immunohistochemical staining using the astrocytic markers GFAP and S100β. In the hippocampal CA1 *str. rad.*, S100β^+^ cell density was not affected by Cx43 overexpression (CTRL: 24422.9 ± 5646.5 vs. Cx43^+^: 27578 ± 7232.5 *p* = 0.33, LMM, Fig. 2A), whereas 3D reconstruction of S100β-positive cells revealed significantly reduced volumes of Cx43^+^ astrocytes compared to CTRL astrocytes (CTRL: 590.7 ± 263.5 µm^3^ vs. Cx43^+^: 367.4 ± 220.1 µm^3^, *p* = 0.008, LMM). In accordance with the altered astrocytic morphology, GFAP immunoreactivity was reduced in Cx43^+^ mice (CTRL: 12.6 ± 2.9 au vs. Cx43^+^: 10 ± 2.1 au, *p* < 0.001, LMM; Fig. 2B, C). Furthermore, 3D reconstructions and cytoskeletal predictions extracted from high-resolution confocal images (Fig. 2D) detected a significant reduction in branch numbers (CTRL: 85.6 ± 48.4 vs. Cx43^+^: 33.9 ± 11.3, *p* = 0.008, LMM) (Fig. 2E), while the ramification index (CTRL: 10.69 ± 1.43 vs. Cx43^+^: 9.4 ± 0.84, *p* = 0.11, LMM) and astrocyte process length (CTRL: 10 ± 0.95 μm vs. Cx43^+^: 10.54 ± 1.6 μm, *p* = 0.22, LMM) remained unchanged (Fig. 2E). Together, these data show that Cx43 overexpression does not affect astrocyte density but their morphology.

Immunohistochemical staining revealed strong mCherry expression not only in astrocytes but also in cells with the morphological characteristics of radial glia (RG)-like cells in the SGZ of the DG (Fig. 2F). These stem cells exhibit glutamate uptake and are coupled through Cx43 GJ channels and loss of coupling in RG-like cells impairs neurogenesis [26, 28]. We therefore investigated whether Cx43 overexpression influences neurogenesis. As adult neurogenesis requires division of RG-like cells, Ki67 immunoreactivity, identifying dividing cells, was quantified in the SGZ of CTRL and Cx43^+^ mice (Fig. 2G). The number of Ki67^+^ cells per section was not different between genotypes (CTRL: 8.3 ± 4.3 vs. Cx43^+^: 6.2 ± 2.4, *p* = 0.26, LMM), indicating that increased Cx43 expression in RG-like cells did not influence adult neurogenesis.

### Cx43 Overexpression Did not Influence Synaptic Plasticity

**Fig. 3:**
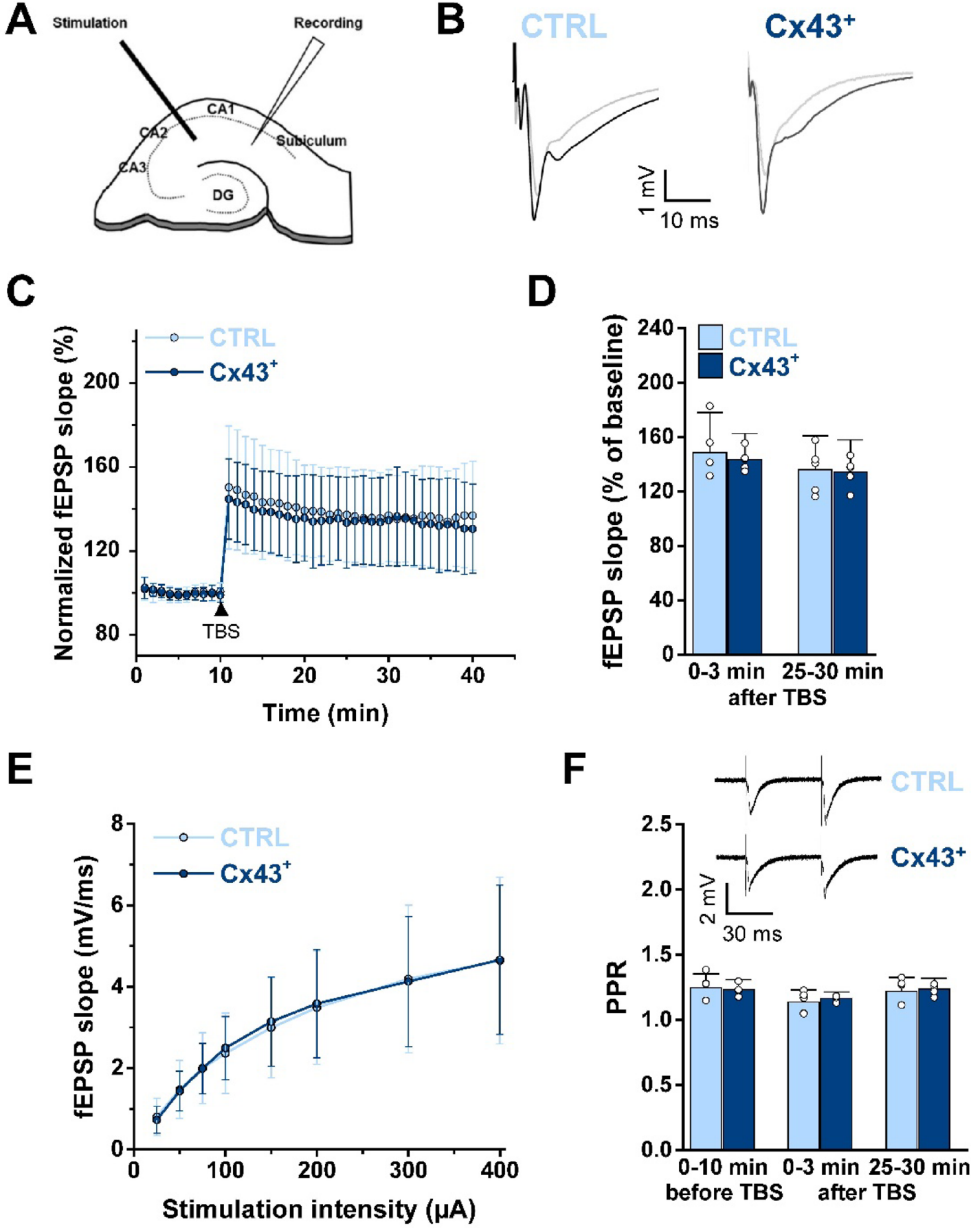
Astrocytic Cx43 overexpression did not affect TBS-induced LTP in the hippocampal CA1 region.**A** Scheme indicating the positions of stimulation and recording electrodes in the CA1 region of the hippocampus. **B** Representative fEPSP traces recorded from Cx43⁺ and CTRL mice before (gray) and 25–30 min after (black) induction of LTP. **C** Time course of normalized fEPSP slopes 10 min before and 30 min after TBS. The magnitude of LTP was not affected by Cx43 overexpression. Data represent mean fEPSP slope per min ± SD, *n* = 15 slices from *N* = 5 animals in each group. **D** Quantification of LTP magnitude recorded during the early (first 3 min) and late (last 5 min) post-TBS periods showed no difference between Cx43⁺ and CTRL mice. *n* = 15 slices from *N* = 5 animals per group. **E** Stimulus-response curves revealed similar fEPSP progression with increasing stimulation intensity, indicating unaffected basal synaptic transmission in Cx43⁺ mice. Data represent mean fEPSP slopes at each stimulus intensity ± SD. *n* = 15 slices from *N* = 5 animals in each group. **F** PPR comparison at baseline (0–10 min before TBS) and during early and late post-TBS periods revealed no differences between groups. Representative traces from a paired-pulse stimulation protocol are shown above the graph. *n* = 15 slices from *N* = 5 animals in each group.

GJ-dependent astrocyte communication is known to play a role in the regulation of synaptic transmission, synaptic plasticity, learning and behavior [23]. To assess whether increased astrocytic Cx43 expression affects synaptic long-term potentiation (LTP), fEPSPs evoked by CA3-CA1 Schaffer collateral stimulation were recorded in the CA1 *str. rad.* of hippocampal slices. LTP was induced by theta-burst stimulation (TBS) following a 10-minute stable baseline, and fEPSP slopes were monitored for 30 min after stimulation (Fig. 3A, B). No significant differences in LTP were observed between Cx43^+^ mice and CTRL mice (0–3 min after TBS: CTRL: 148.65 ± 29.37 vs. Cx43^+^: 143.35 ± 19.39, *p* = 0.77, Mann-Whitney U-test; 25–30 min after TBS: CTRL: 135.88 ± 25.04 vs. Cx43^+^: 134.15 ± 23.76, *p* = 0.85, t-test; Fig. 3C, D). Furthermore, stimulus-response curves, generated by plotting the fEPSP slopes against increasing stimulation intensities (25–400 µA), were not significantly different between the genotypes (*p* = 0.873, two-way repeated measures ANOVA, Fig. 3E), indicating unaltered basal synaptic transmission. The presynaptic release probability also appeared to be unaffected by Cx43 overexpression, as the paired-pulse ratio (PPR) did not differ between genotypes at any time (0–10 min before TBS: CTRL: 1.25 ± 0.1 vs. Cx43+: 1.23 ± 0.08; 3–5 min after TBS: CTRL: 1.14 ± 0.09 vs. Cx43+: 1.17 ± 0.05; 25–30 min after TBS: CTRL: 1.22 ± 0.11 vs. Cx43+: 1.24 ± 0.08, *p* = 0.69, two-way ANOVA, Fig. 3F). Collectively, these data reveal that Cx43 overexpression has no obvious impact on excitatory synaptic transmission or its LTP.

### Cx43 Overexpression Attenuates Chronic Epileptiform Activity

**Fig. 4:**
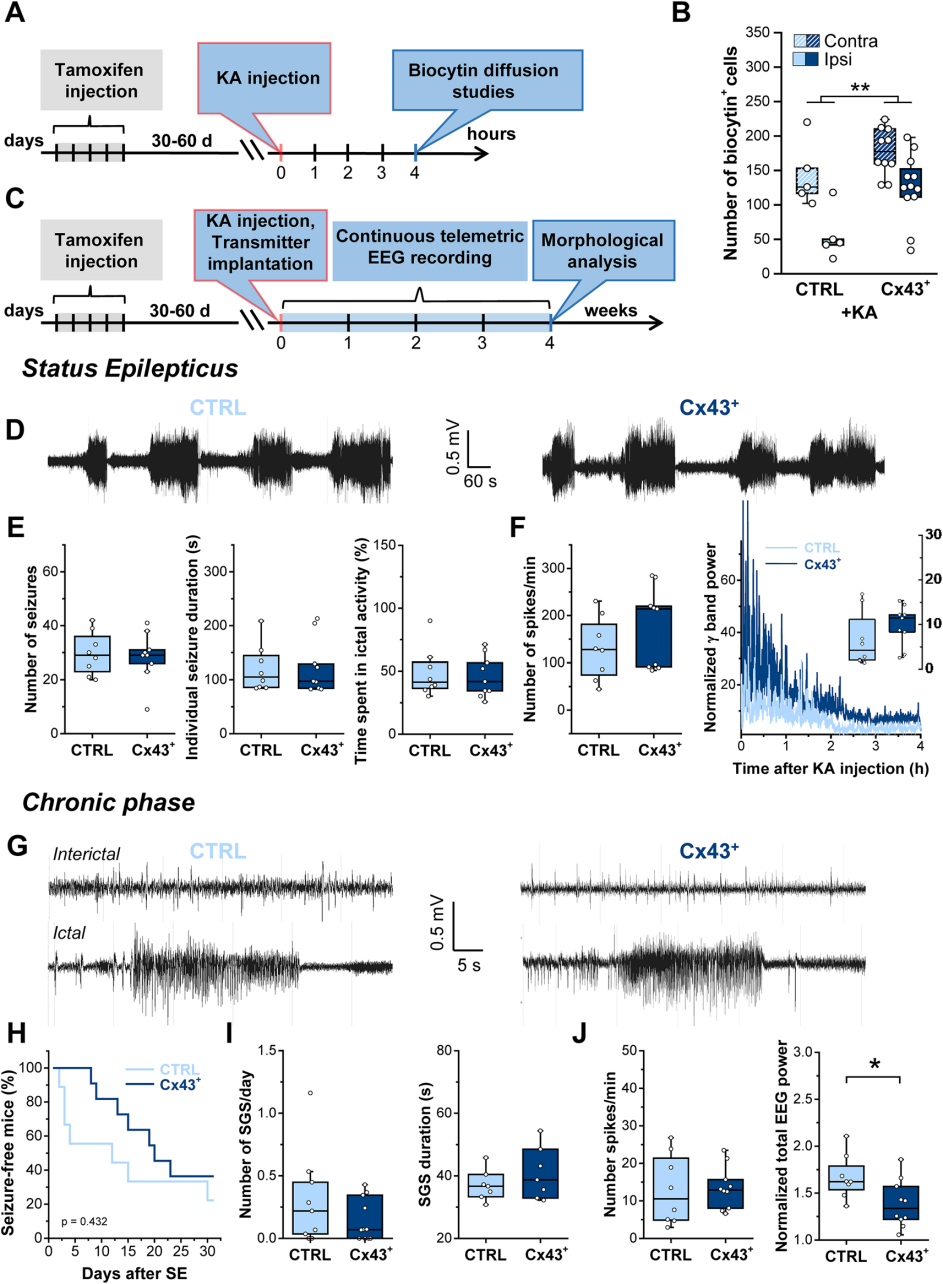
Astrocytic Cx43 overexpression had no effect on SE severity but reduced total EEG power in the chronic phase. **A** Schematic of the experimental procedure for tracer coupling analysis. Cx43^+^ and CTRL mice were i.p. injected with tamoxifen (2 mg/mouse/day) for 5 consecutive days. After 17–22 days, mice received stereotactic unilateral intracortical KA injections. Astrocytic coupling in the ipsi- and contralateral hippocampal CA1 region was assessed via biocytin diffusion studies 4 h after KA-induced SE. **B** Quantification of biocytin diffusion in KA-injected CTRL and Cx43^+^ mice. Both genotypes showed significant ipsilateral reductions in biocytin^+^ astrocytes, yet the overall number of biocytin^+^ cells was significantly greater in Cx43^+^ mice. *n* = 5 and 12 slices from CTRL and Cx43^+^ mice, derived from *N* = 3 and 5 animals, respectively. **C** Schematic of the experimental procedure used for EEG analyses. Immediately after KA injection, tamoxifen-treated Cx43^+^ and CTRL mice were implanted with telemetry transmitters. EEG was then recorded continuously for 4 weeks. Mice were subsequently sacrificed, and hippocampal histopathology was assessed by immunohistochemistry. **D** Representative EEG traces obtained during KA-induced SE in Cx43^+^ and CTRL mice. **E** Number of seizures, seizure duration, and time spent in ictal activity during the first hour of SE. None of these parameters were affected by Cx43 overexpression. **F** Analyses of spike frequency and normalized high frequency EEG activity (γ = 30–50 Hz) during the first 4 h of EEG recording revealed no difference between genotypes (inset displays the median γ band activity over 4 h of SE). *N* = 9 Cx43^+^ and 8 CTRL mice. **G** Representative EEG traces (interictal activity, top; ictal activity, bottom) from Cx43^+^ and CTRL mice during the chronic phase of KA-induced epilepsy. **H** Kaplan–Meier curve showing that the onset of SGS activity was not different between the genotypes. **I** Total number of SGS and their duration did not differ between genotypes. **J** Normalized total EEG power (right graph) but not spike frequency (left graph) was significantly reduced in CX43^+^ mice. *N* = 10 Cx43^+^ and 8 CTRL mice, *p < 0.05, **p < 0.01, ***p < 0.001. Boxplots represent median and quartiles, with whiskers extending to the highest and lowest values within 1.5 × interquartile range. Open circles represent individual data points.

To elucidate effects of Cx43 overexpression on the generation and progression of epilepsy, we subjected Cx43^+^ and CTRL mice to the unilateral intracortical KA model of TLE [14]. Our previous work has demonstrated that a reduction in hippocampal astrocytic coupling occurs as early as 4 h after KA injection, preceding neuronal death and the onset of spontaneous seizures, thus implying a causal role in epileptogenesis [14, 17]. We examined the impact of seizures on astrocytic coupling in Cx43^+^ and CTRL mice by comparing biocytin diffusion after patch clamp recording on the ipsilateral and contralateral side (Fig. 4A). Four h after KA injection, the ipsilateral numbers of biocytin^+^ astrocytes were robustly decreased in both genotypes (CTRL, contra: 143.6 ± 46.6 vs. ipsi: 55.4 ± 36.6 cells; Cx43^+^, contra: 177.4 ± 34.1 vs. ipsi: 125.6 ± 48 cells); however, the overall coupling efficiency Cx43^+^ mice was significantly higher than in CTRL mice (two-way ANOVA, significant effect of the side (contra vs. ipsi, *p* < 0.001) and genotype (CTRL vs. Cx43^+^, *p* = 0.006) but no interaction of the two factors (genotype and side, *p* = 0.37)) (Fig. 4B). Notably, coupling on the ipsilateral side of Cx43^+^ mice was comparable to that on the contralateral side of CTRLs. We triggered SE via intracortical KA injections 30 to 90 days after tamoxifen-induced recombination in Cx43^+^ and CTRL mice, and continuously monitored epileptiform activity during SE and the chronic phase using telemetric EEG recordings (24 h/day) for 4 weeks (Fig. 4C). For quantification of SE we first determined the number and duration of seizures as well as the time spent in ictal activity during the first hour of EEG recording (Fig. 4D, E). This type of quantification was limited to the first hour because individual seizure events can only be accurately identified within this timeframe in this model [17, 18]. There was no difference between Cx43^+^ and CTRL mice in terms of seizure numbers (CTRL: 29.75 ± 8.37 vs. Cx43^+^: 28.44 ± 9.17 seizures/h; *p* = 0.76, t-test), duration of individual seizures (CTRL: 120.5 ± 46.3 s vs. Cx43^+^: 123.69 ± 51.1 s; *p* = 0.7, Mann–Whitney U-test), or time spent in ictal activity (CTRL: 48.75 ± 17.35% vs. Cx43^+^: 45.9 ± 16.4%; *p* = 0.75, t-test). To assess the overall strength of SE, which lasted ~ 4 h [14], EEG data from the first 4 h after KA injection were subjected to spike and spectral analyses. Again, no significant differences were observed between CTRL and Cx43^+^ mice in spike frequency (CTRL: 130.3 ± 65.8 vs. Cx43^+^: 175.25 ± 84.67 spikes/min; *p* = 0.28, Mann–Whitney U-test), normalized high-frequency (γ band) power (CTRL: 6.6 ± 6.2 vs. Cx43^+^: 9.8 ± 4.5; *p* = 0.24, Mann–Whitney U-Test) (Fig. 4F), or normalized total power (CTRL: 6.1 ± 6.3 vs. Cx43^+^: 7.2 ± 4.6; *p* = 0.66, t-test; not shown). Following a comparable latent period (chi-squared = 0.62, *p* = 0.432, log-rank test), 77.78% (7 of 9) CTRL mice and 63.63% (7 of 11) Cx43^+^ mice developed chronic spontaneous generalized seizures (SGS) (Fig. 4G, H), whose frequency and duration, however, did not differ between genotypes (seizure frequency: CTRL: 0.3 ± 0.38 vs. Cx43^+^: 0.14 ± 0.17 seizures/day; *p* = 0.42, Mann-Whitney U-Test; seizure duration: CTRL: 37 ± 4.9 s vs. Cx43^+^: 40.78 ± 8.33 s, *p* = 0.32, t-test) (Fig. 4I). Interestingly, although the frequency of EEG spikes did not differ between genotypes (CTRL: 12.9 ± 9.3 vs. Cx43^+^: 13.45 ± 5.6 spikes/min; *p* = 0.88, t-test), the normalized total power was significantly lower in Cx43^+^ vs. CTRL mice (CTRL: 1.67 ± 0.23 vs. Cx43^+^: 1.38 ± 0.25; *p* = 0.026, t-test) (Fig. 4J). When considering the relevant frequency bands, consistent with the spike-and-wave nature of interictal epileptiform activity (γ for spikes, δ for waves), we further observed a significant reduction in δ-band power (CTRL: 3.1 ± 1.6 vs. Cx43^+^: 1.53 ± 0.3; *p* = 0.009, t-test) in Cx43^+^ mice, whereas γ-band power (CTRL: 1.3 ± 0.3 vs. Cx43^+^: 1.4 ± 0.24; *p* = 0.44, t-test) did not differ between genotypes (data not shown). These data indicate that astrocytic Cx43 overexpression does not affect SE severity but attenuates chronic epileptic activity.

### Cx43 Overexpression Attenuates TLE-associated Sclerosis

**Fig. 5:**
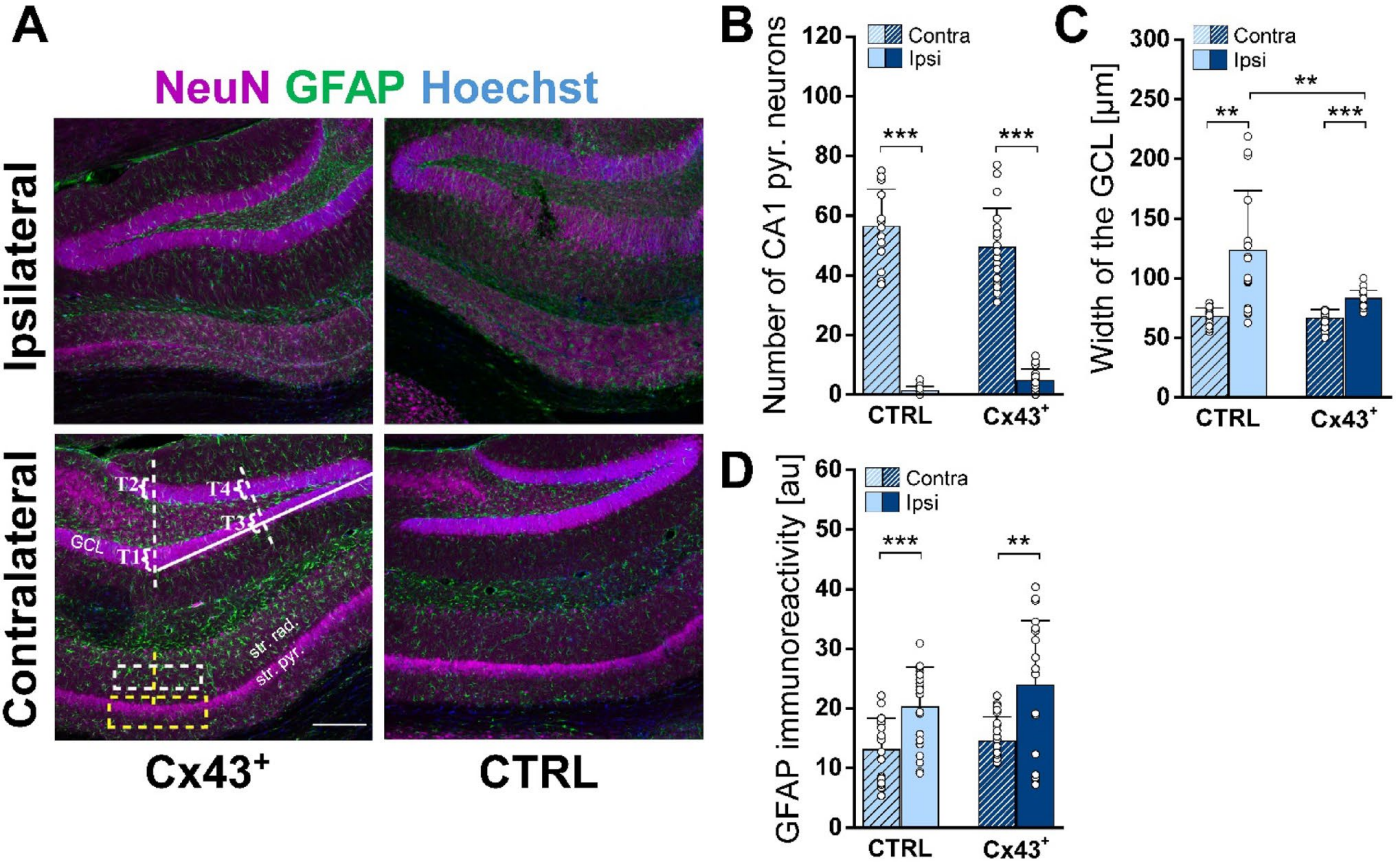
Cx43 overexpression attenuated HS in chronic TLE. **A** Representative MIPs of NeuN (magenta), GFAP (green), and Hoechst (blue) triple staining in ipsi- and contralateral hippocampal slices from Cx43^+^ and CTRL mice obtained 4 weeks after KA injection. Labelling in the lower left image: *str. pyr.*,* stratum pyramidale; str. rad.*,* stratum radiatum; GCL*, granule cell layer; T1-T4, positions at which the GCL width was measured. The white dashed box in the *str. rad.* indicates the ROI (320 × 100 × 23.3 μm³) used for the quantification of astrogliosis, the yellow dashed box in the *str. pyr.* indicates the ROI (360 × 120 × 30 μm³) within which pyramidal neurons were counted. Scale bar: 200 μm. **B–D** KA-injected Cx43⁺ mice displayed similar ipsilateral pyramidal cell loss in the CA1 region and a comparable extent of astrogliosis in the *str. rad.* as CTRL mice. In contrast, ipsilateral GCD was less pronounced in KA-injected Cx43^+^ mice. *n* = 20 and 25 slices from CTRL and Cx43^+^ mice, derived from *N* = 4 and 5 animals, respectively. *p < 0.05, **p < 0.01 ***p < 0.001.

HS, characterized by neuronal loss in the CA1 and CA4 regions, reactive gliosis, and GCD, is a frequent finding in pharmacoresistant TLE patients [29] and is reliably reproduced in our experimental model [14]. To determine whether enhanced astrocytic coupling affects the development of this histopathological alteration, we evaluated the extent of HS after EEG recordings using immunohistochemical staining with antibodies against the neuronal marker NeuN and the astrocytic marker GFAP (Fig. 5A). The extent of neurodegeneration was quantified by counting the number of NeuN^+^ in ROIs of 360 × 120 × 30 μm³ within the CA1 *str. pyr.* on the ipsilateral and contralateral sides. Both genotypes exhibited severe ipsilateral neurodegeneration, which was not significantly different between genotypes (number of neurons: CTRL, contra: 56.4 ± 12.5 vs. ipsi: 1.33 ± 1.44; Cx43^+^, contra: 49.5 ± 12.9 vs. ipsi: 4.74 ± 3.9; LMM interaction between side and genotype, *p* = 0.014; post hoc tests: CTRL ipsi vs. contra, *p* < 0.001; Cx43⁺ ipsi vs. contra, *p* < 0.001; CTRL ipsi vs. Cx43⁺ ipsi, *p* = 0.45) (Fig. 5A, B). Intriguingly, ipsilateral GCD was significantly less pronounced in Cx43⁺ mice compared to CTRL mice (CTRL, contra: 67.9 ± 6.9 μm vs. ipsi: 123.15 ± 50.3 μm; Cx43^+^, contra: 66.3 ± 7.6 μm vs. ipsi: 83 ± 6.6 μm, LMM interaction (side x genotype) *p* < 0.001; post hoc tests: CTRL ipsi vs. contra, *p* = 0.0014; Cx43⁺ ipsi vs. contra, *p* < 0.001; CTRL ipsi vs. Cx43⁺ ipsi, *p* = 0.0035) (Fig. 5A, C). Astrogliosis, assessed by GFAP immunoreactivity within ROIs (320 × 100 × 23.3 μm³) in the *str. rad.* of the CA1 region, was similarly increased ipsilaterally in both CTRL and Cx43^+^ mice (CTRL, contra: 13 ± 5.3 vs. ipsi: 20.2 ± 6.7 au, Cx43^+^, contra 14.5 ± 4.1 vs. ipsi: 23.9 ± 10.8 au; LMM interaction (side x genotype) *p* = 0.64; post hoc tests: CTRL ipsi vs. contra, *p* < 0.0001; Cx43⁺ ipsi vs. contra, *p* = 0.004; CTRL ipsi vs. Cx43⁺ ipsi, *p* = 0.17) (Fig. 5A, D). Taken together, these data indicate that increased astrocytic coupling mitigates the extent of seizure-induced HS, as reflected by the attenuated GCD.

## Discussion

To gain a deeper understanding of the role of the astroglial network under physiological and pathophysiological conditions, we generated a transgenic mouse line with inducible overexpression of Cx43 in astrocytes. In hippocampal slices from these mice, we observed not only increased Cx43 protein levels but also enhanced interastrocytic diffusion of biocytin, indicating that Cx43 overexpression augments the coupling efficiency between astrocytes. The fact that this did not lead to a reduction in membrane resistance or an increase in capacitance can be explained by the limited voltage-clamp control during patch clamp analyses of astrocytes in acute slice preparations. Previous reports have emphasized that in situ, the low glial input resistance and a significant intercellular conductance impede any reliable quantitative analysis of intrinsic astrocytic membrane parameters [11, 30].

While an intact astroglial network can be important for normal neuronal function [8, 9, 11], it remains unclear whether increasing coupling through GJ overexpression provides an additional benefit. Astrocytes are already extensively interconnected under physiological conditions, suggesting that network-mediated functions operate close to their optimal level, leaving little capacity for further improvement. Consistent this, we could not detect any effect of Cx43 overexpression on synaptic efficacy or plasticity. Inhibition of astrocytic coupling by expressing dominant-negative Cx43 impaired hippocampal LTP, which was rescued by lactate application, indicating that metabolic support via astrocytic GJs is critical for synaptic plasticity [10]. Previous studies have shown that during periods of high energy demand, activity-dependent diffusion of energy metabolites through the astroglial network is essential for maintaining hippocampal synaptic transmission [12]. Under physiological conditions, optimal metabolic supply of neurons by the astroglial network is probably already ensured, which may explain why overexpression of Cx43 had no effect. In constitutive Cx30/Cx43 dKO mice, Pannasch et al. (2011) attributed impaired LTP and enhanced synaptic transmission to deficient extracellular buffering of glutamate and K^+^. This would increase synaptic release probability and insertion of postsynaptic AMPARs, which unsilences synapses [8]. In inducible dKO mice the deficits were linked to altered Ca²⁺-driven astrocytic D-serine release and inflammatory cytokines [9]. dKO mice lack Cx43 and Cx30, so it remains unclear whether the observed synaptic changes were due to loss of GJ coupling or the absence of Cx protein. Notably, Cx30 modulates synaptic strength by restricting cleft invasion of glial processes and thus glutamate buffering, independently of its channel function [6]. Interestingly, both deletion and overexpression of Cx30 resulted in reduced synaptic glutamate concentration and impaired LTP [6, 31]. The authors suggest that in Cx30-deficient mice, this effect results from non-channel functions of Cx30 protein, while its overexpression promotes redistribution of glutamate and K^+^ within the astroglial network, thereby increasing the efficiency of astrocytic glutamate uptake. However, differences in conductivity, selectivity, voltage sensitivity, gating properties and regulation between Cx30 and Cx43 limit the applicability of Cx30 overexpression findings to our study. Further work is needed to clarify whether Cx43 overexpression affects the effectiveness of intercellular diffusion of ions, neurotransmitters, or metabolites.

GJ coupling between RG-like cells in the SGZ of the DG has been linked to the regulation of adult neurogenesis [13, 26]. As the name indicates, RG-like cells express various glial proteins, including Cx43, GFAP and GLAST [13, 26, 28]. The high mCherry expression in cells with morphological characteristics of RG-like cells in the DG marks Cx43 overexpression in these cells, which, however, did not seem to affect neurogenesis. How exactly Cxs regulate neurogenesis is unclear, but it has been shown that channel properties of Cx43, not Cx30, are necessary for this function [26]. Again, the ionic and/or metabolic coupling between RG-like cells necessary for adult neurogenesis seems to reach saturation under physiological conditions, so increasing coupling further does not improve the process.

A dysfunctional astroglial network has been linked to the pathogenesis of TLE, but its precise role and mechanisms are not fully understood [14, 15]. Our previous work has shown that a reduction in hippocampal astrocytic coupling occurs early in experimentally induced epilepsy, suggesting a causal role in epileptogenesis [14, 17]. In this study, we replicated the early reduction in astrocytic coupling in CTRL mice. In Cx43 overexpressing mice, uncoupling was less severe, with ipsilateral coupling remaining significantly higher than in controls and comparable to the contralateral side of CTRL animals. Accordingly, these mice serve as a model to study epileptogenesis under conditions of maintained astrocytic coupling.

The disruption of GJ coupling between astrocytes can, in principle, influence neuronal excitability and synchrony in different ways. A major consequence of disrupted glial coupling is a reduced capacity to buffer extracellular K⁺ during intense neuronal activity, leading to its accumulation and, consequently, to enhanced neuronal depolarisation and hyperexcitability [11, 32]. Moreover, glutamate uptake by astrocytic transporters depends on the Na⁺ gradient, and impaired coupling could lead to cytosolic Na⁺ buildup, reducing glutamate clearance and enhancing excitatory transmission [15, 33]. Elevated cytosolic Na⁺ may also cause the Na⁺/Ca²⁺ exchanger to reverse, resulting in astrocytic Ca²⁺ influx and impaired Ca²⁺-dependent processes like aberrant gliotransmitter release, which in turn affects network excitability [34, 35]. Finally, the accumulation of cytosolic ions may cause astrocyte swelling, which decreases the extracellular space and intensifying the accumulation of ions and neurotransmitters, thereby increasing seizure susceptibility [8, 15]. Despite strong evidence for an antiseizure role, the activity-dependent transfer of energetic metabolites from blood vessels to areas of high demand seems necessary to sustain (rather than initiate) seizure activity [12]. Thus, it was suggested that an acute reduction in astrocytic GJ coupling may have immediate seizure-promoting effects due to decreased K^+^ and glutamate buffering, but delayed seizure-suppressing effects due to insufficient energy supply [15]. Studies on Cx30/Cx43 dKO mice have not provided a clear picture of the role of astrocytic GJs in epilepsy, as some reported spontaneous epileptiform activity in hippocampal slices and increased seizure susceptibility in a chronic TLE model [11, 18], while another study showed reduced sensitivity to PTZ-induced hyperexcitability [19]. As mentioned above, dKO mice – unlike the situation in human TLE-HS is - lack not only intercellular coupling but also the Cx protein, so that hemichannels can’t form and non-channel functions of Cxs are lacking [16]. This limitation does not apply to Cx43^+^ mice, where coupling and protein levels were increased. Our data show that, while KA-induced SE severity remained unaffected, in the long term overexpression of Cx43 attenuated chronic EEG power and the extent of HS, consistent with the view that the astroglial network plays an antiepileptic role.

Overall, our data suggest that in the healthy brain, the functions mediated by the astroglial network are optimally maintained and cannot be further enhanced by increasing coupling. The observation that Cx43 overexpressing mice display reduced epileptiform EEG activity and less pronounced histopathological changes supports the view that the loss of astrocytic coupling, as seen in human and experimental MTLE-HS, contributes to disease pathogenesis. Importantly, the present findings provide new insights into the role of the GJ–coupled astroglial network in the healthy and diseased brain, which may prove valuable for the development of more effective antiepileptic therapies.

## Data Availability

The datasets generated during and/or analysed during the current study are available from the corresponding author on reasonable request.
