# Correction: Induced Overexpression of Connexin43 in Astrocytes Attenuates the Progression of Experimental Temporal Lobe Epilepsy

**DOI:** 10.1007/s11064-025-04572-y

**Published:** 2025-10-10

**Authors:** Oussama Kherbouche, Lukas Henning, Pia Niemann, Caroline Geisen, Gerald Seifert, Christian Henneberger, Bernd K. Fleischmann, Christian Steinhäuser, Peter Bedner

**Affiliations:** 1 Institute of Cellular Neurosciences I, Medical Faculty, University of Bonn, Bonn, Germany; 2 Institute of Physiology I, Medical Faculty, University of Bonn, Bonn, Germany; 3 German Center for Neurodegenerative Diseases (DZNE), Bonn, Germany; 4 Present Address: Klinik für Neurologie, KRH Klinikum Agnes Karll Laatzen, Hannover, Germany; 5 Present Address: Department of Epileptology, University Hospital Bonn, Bonn, Germany

**Correction to: Neurochemical Research (2025) 50:303** 10.1007/s11064-025-04558-w

In this article Fig. 4 appeared incorrectly and have now been corrected in the original publication. For completeness and transparency, the correct and old incorrect versions are displayed below.

The original article has been corrected.

Incorrect version


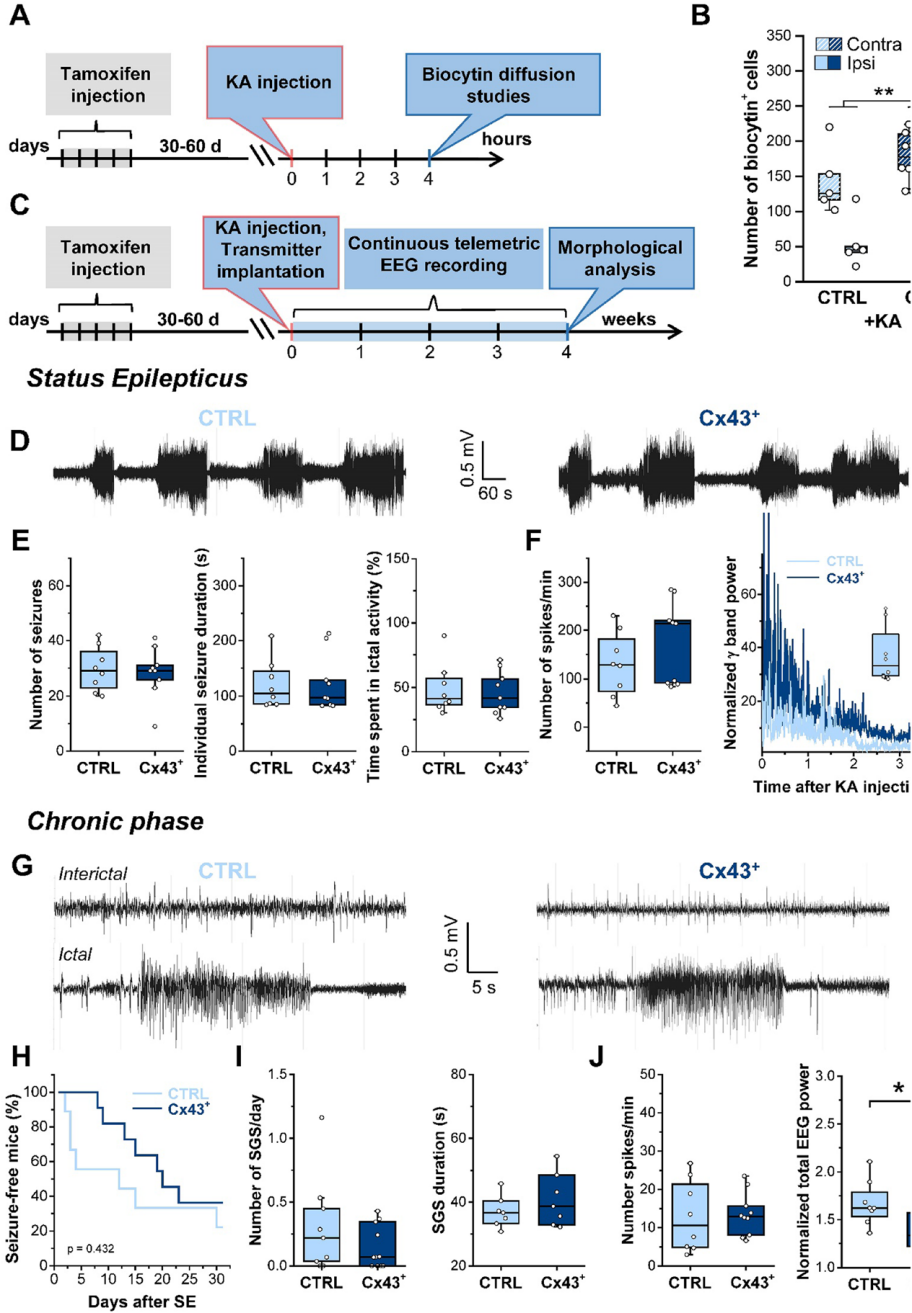


Correct version of Fig. 4

**Fig. 4:**
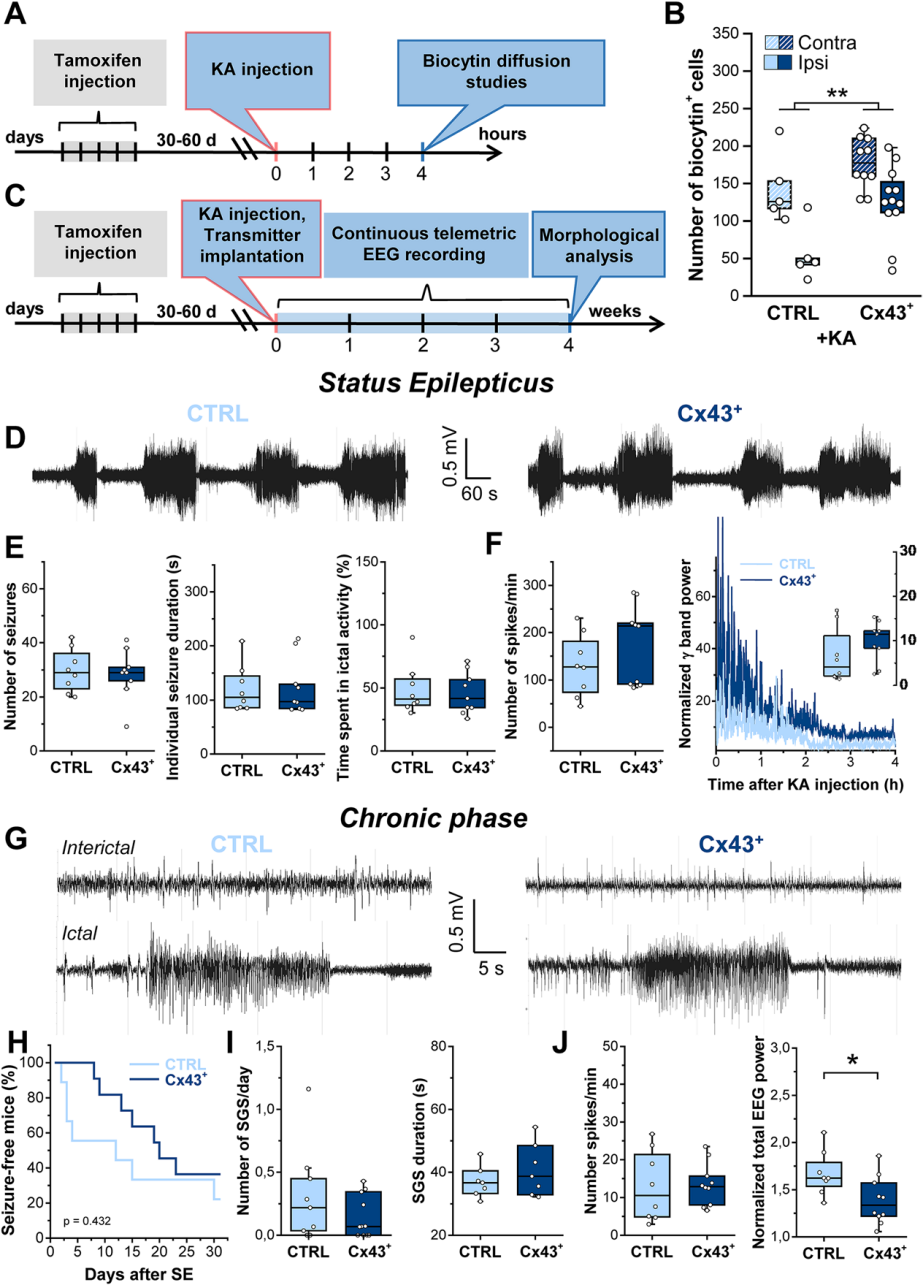
Astrocytic Cx43 overexpression had no effect on SE severity but reduced total EEG power in the chronic phase. **A** Schematic of the experimental procedure for tracer coupling analysis. Cx43^+^ and CTRL mice were i.p. injected with tamoxifen (2 mg/mouse/day) for 5 consecutive days. After 17–22 days, mice received stereotactic unilateral intracortical KA injections. Astrocytic coupling in the ipsi- and contralateral hippocampal CA1 region was assessed via biocytin diffusion studies 4 h after KA-induced SE. **B** Quantification of biocytin diffusion in KA-injected CTRL and Cx43^+^ mice. Both genotypes showed significant ipsilateral reductions in biocytin^+^ astrocytes, yet the overall number of biocytin^+^ cells was significantly greater in Cx43^+^ mice. *n* = 5 and 12 slices from CTRL and Cx43^+^ mice, derived from *N* = 3 and 5 animals, respectively. **C** Schematic of the experimental procedure used for EEG analyses. Immediately after KA injection, tamoxifen-treated Cx43^+^ and CTRL mice were implanted with telemetry transmitters. EEG was then recorded continuously for 4 weeks. Mice were subsequently sacrificed, and hippocampal histopathology was assessed by immunohistochemistry. **D** Representative EEG traces obtained during KA-induced SE in Cx43^+^ and CTRL mice. **E** Number of seizures, seizure duration, and time spent in ictal activity during the first hour of SE. None of these parameters were affected by Cx43 overexpression. **F** Analyses of spike frequency and normalized high frequency EEG activity (γ = 30–50 Hz) during the first 4 h of EEG recording revealed no difference between genotypes (inset displays the median γ band activity over 4 h of SE). *N* = 9 Cx43^+^ and 8 CTRL mice. **G** Representative EEG traces (interictal activity, top; ictal activity, bottom) from Cx43^+^ and CTRL mice during the chronic phase of KA-induced epilepsy. **H** Kaplan–Meier curve showing that the onset of SGS activity was not different between the genotypes. **I** Total number of SGS and their duration did not differ between genotypes. **J** Normalized total EEG power (right graph) but not spike frequency (left graph) was significantly reduced in CX43^+^ mice. *N* = 10 Cx43^+^ and 8 CTRL mice, *p < 0.05, **p < 0.01, ***p < 0.001. Boxplots represent median and quartiles, with whiskers extending to the highest and lowest values within 1.5 × interquartile range. Open circles represent individual data points

